# APS *Data Management System*

**DOI:** 10.1107/S1600577518010056

**Published:** 2018-08-15

**Authors:** Siniša Veseli, Nicholas Schwarz, Collin Schmitz

**Affiliations:** a Argonne National Laboratory, 9700 South Cass Avenue, Argonne, IL 60439, USA

**Keywords:** computer software, data management, data processing

## Abstract

As the capabilities of modern X-ray detectors and acquisition technologies increase, so do the data rates and volumes produced at synchrotron beamlines. This brings into focus a number of challenges related to managing data at such facilities, including data transfer, near real-time data processing, automated processing pipelines, data storage, handling metadata and remote user access to data. The Advanced Photon Source *Data Management System* software is designed to help beamlines deal with these issues. This paper presents the system architecture and describes its components and functionality; the system’s current usage is discussed, examples of its use have been provided and future development plans are outlined.

## Abstract 2

A description of the *Data Management System* developed at the Advanced Photon Source is given.

## 1. Introduction   

Specific data management requirements typically vary from beamline to beamline, generally dependent upon the type of detectors, X-ray techniques and data processing tools in use. However, most of these requirements are related to a set of tasks that are common to all synchrotrons and other large data-generating scientific user facilities:

(i) Storage area management, *i.e.* movement of acquired data from local (short-term) storage to a more permanent location (medium/long-term storage).

(ii) Enabling users and applications to easily find and access data. This includes the use of metadata, replica catalogs and remote data access tools.

(iii) Facilitating data processing and analysis with automated or user-initiated processing workflows.

User facilities have often developed internal systems to address their specific needs. The Advanced Light Source and its collaborators have worked to deploy the *SPOT* suite of data management tools at a number of beamlines. Datasets are transferred to the National Energy Research Scientific Computing Center after collection, where they are archived on an HPSS tape system. Some automated data processing may be launched as data are collected. Metadata are added to a MongoDB-based catalog and users can access their data *via* a web portal (Parkinson *et al.*, 2016 ▸; Blair *et al.*, 2014 ▸; Deslippe *et al.*, 2014 ▸). The LCLS has developed and deployed a uniform infrastructure for data management across all of its experiment end-stations. The infrastructure includes a tiered storage scheme and fast computational resources. A web portal allows users and staff to manage storage and to retrieve data (Thayer *et al.*, 2016 ▸; Damiani *et al.*, 2016 ▸). The NSLS-II records and manages data with the *databroker* tool. Data I/O details are abstracted away by the *databroker*, which exposes data to the user *via numpy* arrays in Python. Data at the NSLS-II reside on either central or beamline local storage systems (Arkilic *et al.*, 2017 ▸).

Until recently, beamlines at the Advanced Photon Source (APS) had no other option but to handle some of the above tasks independently, and simply ignore those that would require too much effort or demand technical skills that beamline scientists might not possess. As a result, the extent of various data-management related services provided by APS beamlines to their users varied greatly from beamline to beamline. For example, very few beamlines provided their users with any kind of remote access to their data, no beamlines had a framework for automated raw data processing and no beamlines kept track of user data or utilized searchable metadata catalogs. In addition, any tools that beamline scientists typically used for managing data were mostly based on ‘*ad hoc*’ beamline-specific scripts that could not be reused on other beamlines without significant additional effort.

The APS *Data Management System* (*DM*) initially had a single objective: provide APS users with means to easily access their data remotely. Over time a number of new features and services were added to the system. New development was and still is driven by the increasing number of user requests and new beamline deployments. Today the *DM* software serves over 20 APS end-stations, and new ones are added to the system fairly regularly.

In the following sections we discuss the system architecture and features, describe its usage, and present plans for future enhancements.

## 2. System components and functionality   

**Figure 1:**
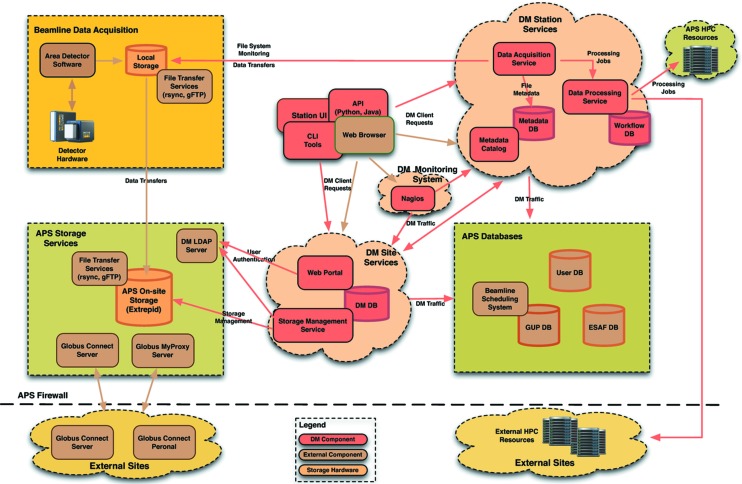
APS *Data Management System* architecture.

As illustrated on the system-architecture diagram (see Fig. 1 ▸), the *DM* software consists of several site-wide and beamline-specific services that handle data movement and metadata cataloging, manage user workflows and processing jobs, as well as manage APS storage and user data access. These services interact with various APS databases and are supported by Globus (https://www.globus.org; Globus toolkit documentation and downloads can be found at http://toolkit.globus.org/toolkit) and other open-source software components.

The system currently uses a 1.5 PB data-direct-networks (DDN) storage (http://www.ddn.com) with a high-performance GPFS file system (Schmuck & Haskin, 2002 ▸) and two 10 Gbps network links. The storage has data redundancy enabled. Read-only data access is provided *via* the ‘aps#data’ Globus endpoint, while user authentication is handled by the Globus MyPr­oxy and *DM* LDAP servers. User authorization to access experimental data files is based on experiment LDAP group membership. The *DM* system allows for a straightforward integration with any (external or internal) computing facility *via* its workflow management functionality. At present, users are regularly processing their data using the APS 1440-core (2880 hyper-threads) high-performance computing (HPC) cluster.

### 2.1. *DM* site services   

*DM* site-wide services are backed by the PostgreSQL (https://www.postgresql.org) *DM* database that contains user information and *DM* experiment metadata. The term ‘experiment’, in this case, represents an arbitrary name that is associated with a collection of data files. For APS beamlines, a *DM* experiment typically corresponds to a set of measurements carried out under one general user proposal (GUP) or under one experiment safety authorization form (ESAF). The main *DM* database is regularly synchronized with the central APS user database. The synchronization is fully automated; it involves adding new users and updating existing user information in the *DM* database, as well as updating user passwords stored in the *DM* LDAP server. By interfacing with the APS user database, the *DM* software reduces to a minimum of its overhead in terms of user management. It is worth emphasizing that the system is designed to be entirely self-sufficient, and hence it also supports managing and maintaining users directly in the main *DM* database.

The *Storage Management Service* runs on the storage head node and is responsible for managing and providing access to *DM* experimental data directories and files. For example, it is notified whenever a new file is transferred into storage and ensures proper permissions are applied to that file, which enables remote access to data for authorized users. The *Storage Management Service* is implemented in Python (http://www.python.org); it uses the CherryPy web framework (http://cherrypy.org) for providing a RESTful (Fielding, 2000 ▸) web service interface based on the JSON data-interchange format (http://json.org) over the HTTPS protocol. The service also utilizes the SQLAlchemy toolkit (https://www.sqlalchemy.org) as the object-relational mapper for accessing the underlying database.

**Figure 2:**
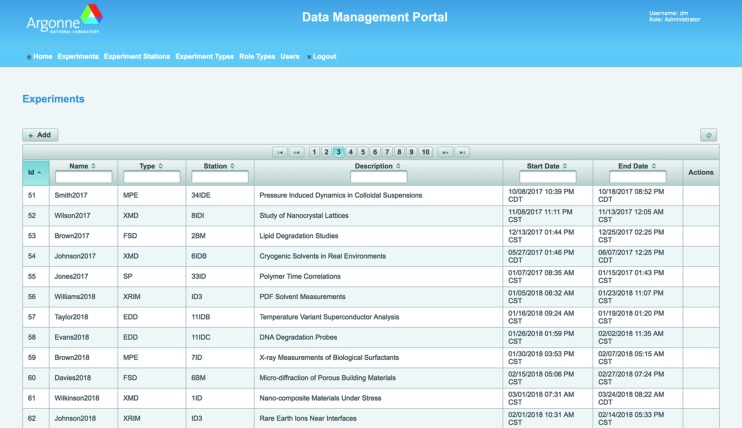
*DM* web portal.

The *DM* web portal (shown in Fig. 2 ▸) provides administrative interfaces for managing user roles, experiments and experiment types, as well as for enabling and supporting beamline deployments. It is implemented as a Java EE application running in a GlassFish application server (https://glassfish.java.net) and built using Java Persistence API (JPA), Java Server Faces (JSF) (http://www.oracle.com/technetwork/java/javaee/overview/index.html) and PrimeFaces component suite (http://primefaces.org).

### 2.2. *DM* station services   

*DM* data management software deployment on each beamline (‘*DM* Station’) includes several Python RESTful web services built on the same set of technologies (Python; CherryPy; JSON; Fielding, 2000 ▸) used by the *Storage Management Service*.

The *Data Acquisition Service* implements a flexible plugin-based processing framework. Its primary role is to monitor local beamline storage during user experiments and initiate file transfers of experimental data to the APS on-site storage. However, it can also be customized to serve the needs of the particular beamline on which it is deployed; this can be done by either configuring the service to use some of the existing processing plugins, or by developing new plugins that fit beamline needs. For example, all beamlines use the same file cataloging plugin, but on some beamlines this plugin is configured to use a custom executable for reading file metadata; similarly, all beamlines use a plugin for transferring data to APS on-site storage. But several beamlines also use additional file transfer plugins that are configured to move data to other destinations, such as internal or external HPC clusters. In most cases, the *Data Acquisition Service* uses GridFTP for file transfers, but can also use rsync utility if GridFTP is unavailable.

File metadata is stored in the beamline metadata catalog, which is backed by MongoDB (https://www.mongodb.com). In addition to user- and experiment-specific information, the system also records information like file size, checksum, processing time and a list of file replicas in the *DM*-managed storage. The metadata can be accessed, updated and deleted *via* the *DM* cataloging service REST interfaces. It can also be viewed *via* the Mongo Express application (Mongo Express releases can be found at GitHub: https://github.com/mongo-express/mongo-express) that runs on the Node.js JavaScript runtime environment (https://nodejs.org) and is served by the Nginx web server.

The *DM* *Processing Service* provides support for managing user-defined workflows as well as for submitting and monitoring user processing jobs based on those workflows. Workflow definitions are kept in the beamline workflow database (MongoDB). A *DM* workflow is defined as a set of processing steps that are executed in order; each step involves an arbitrary command or a script. Whenever possible, the service execution engine takes care of parallelizing the execution of processing steps. The system also supports both input and output arguments in workflow definitions. Input arguments are provided by users or by the system itself (*e.g.* input file paths) whereas output variables are defined as regular expressions that are expected to match the processing step output. Any output variables found for a given step can be used as input arguments for any of the subsequent processing steps in the workflow.

**Figure 3:**
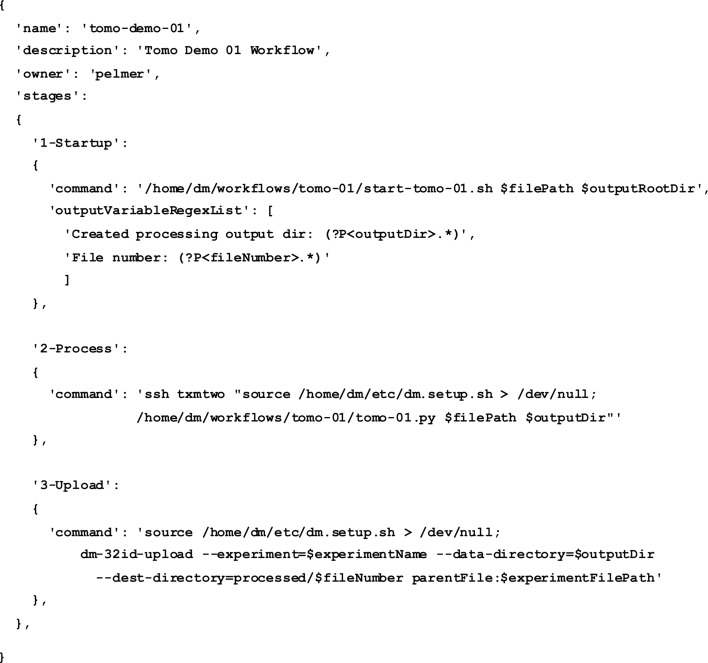
Sample *DM* workflow definition file.

Fig. 3 ▸ illustrates these features by showing the definition of a simple three-stage processing workflow that defines the following stages:

(i) Run the workflow start-up script for a given input file; as a result of the script’s output, two new variables are defined and used in the second are third stages.

(ii) Process the input file on a designated remote machine.

(iii) Upload the processing results to APS on-site storage.

The workflow definition itself is, in this case, embedded within a Python dictionary. The system also supports workflow definitions in the JSON format.

The *Processing Service* can be either used as a standalone, or together with other *DM* station services in support of fully automated beamline data acquisition and processing pipelines. For example, the *Data Acquisition Service* can be configured with an integration plugin that submits user workflow-based jobs to the *Processing Service*. In addition to its REST interfaces, the *Processing Service* offers a ZeroMQ (http://zeromq.org) job submission interface suitable for integration with other beamline applications or software systems.

### 2.3. *DM* monitoring infrastructure   

**Figure 4:**
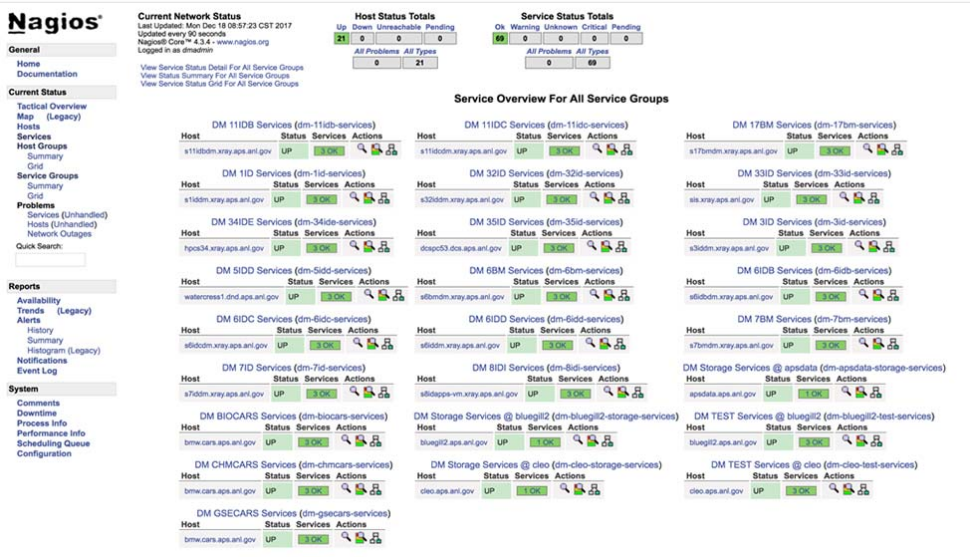
*DM* nagios service groups view.

Every *DM* service has built-in monitoring interfaces that enable external applications to find out about its state. These interfaces are used by custom Nagios (https://www.nagios.org) plugins to provide up-to-date information about the health of all *DM* station deployments. Just like the beamline metadata catalog pages, *DM* Nagios web content is served by the Nginx server (http://nginx.org) (see Fig. 4 ▸).

### 2.4. *DM* user interfaces   

Users and administrators can access the *DM* web portal, *DM* Nagios web pages or beamline metadata catalog pages using their web browsers.

The *DM* REST web service interfaces are accessible programmatically *via* Python and Java APIs. The system also offers a rich set of command-line interfaces and tools that are built on top of its Python APIs. All command-line interfaces are fully scriptable; they share a common base, which results in a common look and feel. User access to REST interfaces is session based, and all *DM* command-line tools take advantage of this feature.

**Figure 5:**
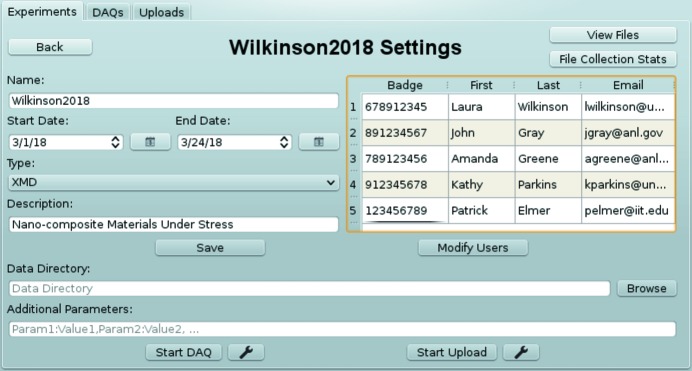
*DM* station GUI.

In addition to command-line tools and APIs, *DM* users also have the option to use the *DM* station GUI, which is implemented using the PyQt toolkit (https://riverbankcomputing. com/software/pyqt/intro). The GUI allows users to create experiments (either manually, or using GUP or ESAF data), modify experiment users, start monitoring data directories for new files or upload existing files to storage. It also has the ability to display experiment-file collections, as well as individual file metadata. The GUI experiment management page is shown in Fig. 5 ▸.

**Figure 6:**
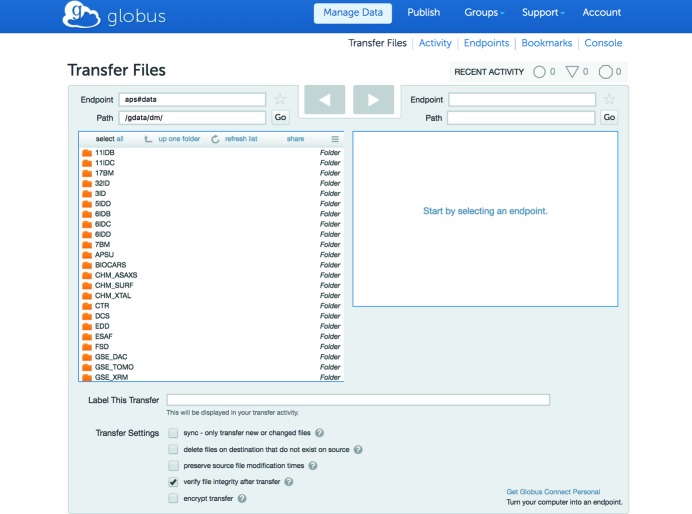
Access to APS data *via* *Globus online*.

APS onsite users can either access their data by using the *DM* download command-line utility, or by using *Globus Online* (see Fig. 6 ▸). The latter option requires *Globus Connect Personal* or *Globus Connect Server* software (https://www.globus.org). Offsite users must always use *Globus Online* for transferring their files to a personal laptop or to a remote site. The rights to use data collected at the APS are governed by user agreements between the operator of the Argonne National Laboratory for the US Department of Energy and users of the facility. In most non-proprietary user agreements, the users of the facility and the US Government have rights to utilize the data.

## 3. Software installation   

Each beamline at the APS has its own data management software installation visible to all beamline computers. In addition to the *DM* software, the deployment area also contains support software packages, beamline databases and configuration files, as well as various runtime and log files. In this way all beamlines are fully independent of each other, which works well in the APS environment where beamline machines typically have different maintenance cycles. The *DM* software has fully scripted installation, upgrade and deployment testing processes, which reduces the maintenance overhead to a minimum, owing to independent beamline software installations.

*DM* services typically run on a designated beamline server machine, which can be either virtual or physical. All services are controlled *via* a standard set of control scripts suitable for the RHEL operating system used at the APS.

Even though the *DM* software was intended for use at the APS, it has been developed in a way that does not prohibit its installation at different sites. The basic software functionality does not depend on anything other than a set of support software packages that can be replicated anywhere, as well as on its configuration files containing URLs to various support services, which are also not site-specific (*e.g.* LDAP, MongoDB, GridFTP, *etc*.). All APS-specific modules, such as those that enable integration with various APS databases, can be disabled or replaced with new modules that provide equivalent functionality.

## 4. System usage   

**Figure 7:**
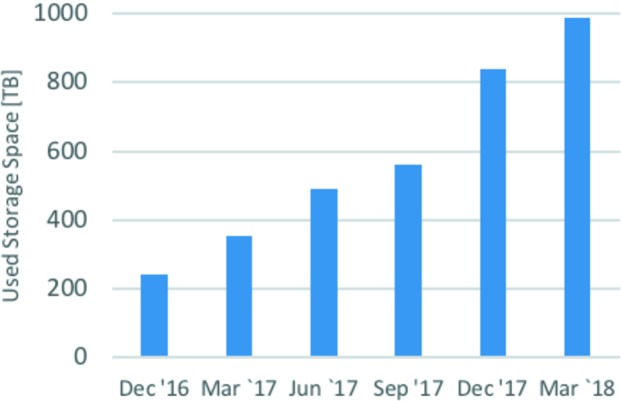
APS on-site storage usage.

The number of *DM* station deployments grew from five in December 2016, to 21 in December 2017, with five new installations coming online in December 2017 alone. The increase in the number of system users is reflected in the corresponding increase in usage of the APS on-site storage, illustrated in Fig. 7 ▸. Since December 2016, the system storage-space usage went from slightly over 250 TB to almost 1 PB (the total storage capacity is 1.5 PB). We should note that the APS storage has data redundancy enabled in order to minimize the possibility of data loss, effectively doubling the size of user data.

One example of the system’s automated processing capability is found in a workflow used by the 8-ID beamline. The APS 8-ID beamline applies the X-ray photon correlation spectroscopy (XPCS) technique to the studies of equilibrium fluctuations and fluctuations about the evolution to equilibrium in condensed matter in the small-angle X-ray scattering (SAXS) geometry. This beamline uses *SPEC* software (https://certif.com/spec.html) for instrument control and data acquisition. For every raw data file, a *SPEC* script starts a *DM* processing job based on the following workflow:

(i) Run a custom shell script to prepare the processing environment (this includes creating the data output directory and determining the appropriate batch queue and job name).

(ii) Copy the raw datafile to the APS HPC cluster using GridFTP (the *globus-url-copy* command).

(iii) Append XPCS metadata to the datafile by running a custom 8-ID-I utility.

(iv) Submit a processing job to the SGE batch scheduler (https://arc.liv.ac.uk/trac/SGE) *via* the *qsub* command. This job runs a custom 8-ID-I processing executable.

(v) Monitor the batch job by running a shell script that interacts with SGE *via* the *qacct* command.

(vi) Copy the resulting output file into the designated beamline storage area using GridFTP (the *globus-url-copy* command).

The APS 8-ID-I beamline may have tens of active *DM* processing jobs running at any given time during a user’s experiment. Those jobs are running on the APS HPC cluster and are monitored by staff and users *via* static web pages generated by a cron job running *DM* utilities.

## 5. Future plans   

The *Data Management* software development is, to a large extent, driven by the needs of beamline users, as well as by the improvements needed to enhance the system’s long-term maintainability and reduce operational overheads. Detailed below are some of the software features planned for the future:

(i) Enable beamline managers to organize their experimental data in storage in a manner that best fits their beamline. Currently all experiment directories in APS storage are contained directly under the top-level beamline data folder. This scheme does not necessarily correspond to the directory structure maintained by beamlines in their local storage areas.

(ii) Further develop functionality offered in the *DM* station GUI (*e.g.* improve file metadata and collection views and add workflow and processing job management capabilities).

(iii) Develop *Data Acquisition Service* plugins that handle integration with external cataloging and data publishing systems, such as the DOE Data Explorer (https://www. osti.gov/dataexplorer/) and the Materials Data Facility (Blaiszik *et al.*, 2016 ▸). Assign persistent digital object identifiers (DOIs) to datasets utilizing the DOE Office of Scientific and Technical Information’s DOI minting service (https://www.osti.gov/data-services).

(iv) Enhance *DM* system monitoring infrastructure by: (*a*) Developing service capabilities for self-diagnosing error or warning conditions and issuing alarms and (*b*) improve support for measuring performance (*e.g.* data-transfer rates, file processing rates, *etc*.).

(v) Further develop beamline management functionality available in the *DM* web portal (*e.g.* add the ability to check on the status of ongoing uploads and initiate new data uploads).

(vi) Develop a standard set of workflow definitions that can be reused on different beamlines for automating processing pipelines.

(vii) Develop a policy engine for automated management of experimental data in storage, archiving of old data, *etc*.

In addition to the above software improvements, the system will also require upgrades on the hardware side: additional storage space, archival system, additional hypervisors for hosting *DM* virtual machines, *etc*. The addition of an archiving functionality will also necessitate developing clear APS-wide policies regarding the length of time for which the old experimental data will be stored.

## 6. Conclusions   

Issues related to beamline data management are not new, but are getting more and more challenging with the ever-increasing rates and volumes of data produced at X-ray facilities. The APS *Data Management System* was started with the goal of enabling offsite users the ability to access their data remotely. In recent years, the system has experienced significant growth in terms of both its usage and capabilities. In this paper, we described the system architecture and current functionality. We also discussed its usage and presented plans for future developments.

## References

[bb1] Arkilic, A., Allan, D. B., Caswell, T. A., Li, L., Lauer, K. & Abeykoon, S. (2017). *Synchrotron Radiat. News*, **30**(2), 44–45.

[bb2] Blair, J., Canon, R. S., Deslippe, J., Essiari, A., Hexemer, A., MacDowell, A. A., Parkinson, D. Y., Patton, S. J., Ramakrishnan, L., Tamura, N., Tierney, B. L. & Tull, C. E. (2014). *Proc. SPIE*, **9212**, 92121G.

[bb3] Blaiszik, B., Chard, K., Pruyne, J., Ananthakrishnan, R., Tuecke, S. & Foster, I. (2016). *JOM*, **68**, 2045–2052.

[bb4] Damiani, D., Dubrovin, M., Gaponenko, I., Kroeger, W., Lane, T. J., Mitra, A., O’Grady, C. P., Salnikov, A., Sanchez-Gonzalez, A., Schneider, D. & Yoon, C. H. (2016). *J. Appl. Cryst.* **49**, 672–679.

[bb5] Deslippe, J., Essiari, A., Patton, S. J., Samak, T., Tull, C. E., Hexemer, A., Kumar, D., Parkinson, D. & Stewart, P. (2014). *Proceedings of the 9th Workshop on Workflows in Support of Large-Scale Science (Works2014)*, 16–21 November 2014, New Orleans, LA, USA, pp. 31–40.

[bb6] Fielding, R. T. (2000). PhD thesis, University of California and Irvine, USA.

[bb7] Parkinson, D. Y., Beattie, K., Chen, X., Correa, J., Dart, E., Daurer, B. J., Deslippe, J. R., Hexemer, A., Krishnan, H., MacDowell, A. A., Maia, F. R. N. C., Marchesini, S., Padmore, H. A., Patton, S. J., Perciano, T., Sethian, J. A., Shapiro, D., Stromsness, R., Tamura, N., Tierney, B. L., Tull, C. E. & Ushizima, D. (2016). *AIP Conf. Proc.* **1741**, 050001.

[bb8] Schmuck, F. & Haskin, R. (2002). *Proceedings of the FAST’02 Conference on File and Storage Technologies*, 28–30 January 2002, Monterey, CA, USA, p. 231.

[bb9] Thayer, J., Damiani, D., Ford, C., Gaponenko, I., Kroeger, W., O’Grady, C., Pines, J., Tookey, T., Weaver, M. & Perazzo, A. (2016). *J. Appl. Cryst.* **49**, 1363–1369. 10.1186/s40679-016-0037-7 PMC5313569 28261541

